# A Latent Class Analysis of Health Characteristics Among Non-Drinking Middle-Aged and Older Adults

**DOI:** 10.1093/geroni/igaf122.2510

**Published:** 2025-12-31

**Authors:** Sara Miller, Jennifer Maggs, Stephanie Lanza

**Affiliations:** The Pennsylvania State University, University Park, Pennsylvania, United States; The Pennsylvania State University, University Park, Pennsylvania, United States; The Pennsylvania State University, University Park, Pennsylvania, United States

## Abstract

Despite documented health consequences of heavy alcohol consumption, there is ongoing debate around potential health benefits of low-to-moderate alcohol use. Better understanding variability in health characteristics among non-drinkers can inform discourse around the alcohol j-curve and underscore the diverse health profiles of adults who abstain in later life. The current study aimed to identify latent classes of non-drinkers based on subjective and objective health characteristics and examine person-level predictors of class membership. Participants were adults aged 50+ years in the Midlife in the United States study who did not report past-month alcohol use (N = 1,205; range=50-84 years). Dichotomous health indicators included any functional impairment, obesity, cardiometabolic risk factors (diabetes, high blood pressure, or stroke), chronic pain, chronic sleep problems, self-reported physical health (1=Fair/Poor; 2=Excellent/Very Good/Good), perceived health in comparison to others (1=Somewhat Worse/Much Worse, 2=Much Better/Somewhat Better/About the Same), chronic depression and/or anxiety, and self-reported mental health (1=Fair/Poor; 2=Excellent/Very Good/Good). Person-level predictors included age (1=Older Adult, 0=Middle-Aged), sex (1=Male, 0=Female), alcohol use history (1=Lifetime Abstainer, 0=Former Drinker), and education (1=College Degree or Higher, 0=Less Than College Degree). Five latent classes were identified: poor overall health (5.9%), poor physical and self-reported health but good mental health (17.9%), poor physical health but good self-reported and mental health (28.5%), good self-reported health but poor physical and mental health (9.8%), and good overall health (38.0%). Age, education, and biological sex, but not alcohol use history, predicted probability of class membership. Future research can explore how the underlying latent classes predict distal health outcomes.

